# Designing persuasive health materials using processing fluency: a literature review

**DOI:** 10.1186/s13104-017-2524-x

**Published:** 2017-06-08

**Authors:** Tsuyoshi Okuhara, Hirono Ishikawa, Masahumi Okada, Mio Kato, Takahiro Kiuchi

**Affiliations:** Department of Health Communication, School of Public Health, The University of Tokyo, 7-3-1 Hongo, Bunkyo-ku, Tokyo, 113-8655 Japan

**Keywords:** Processing fluency, Health literacy, Health information, Health materials, Health education, Readability, Persuasiveness

## Abstract

**Background:**

Health materials to promote health behaviors should be readable and generate favorable evaluations of the message. Processing fluency (the subjective experience of ease with which people process information) has been increasingly studied over the past decade. In this review, we explore effects and instantiations of processing fluency and discuss the implications for designing effective health materials. We searched seven online databases using “processing fluency” as the key word. In addition, we gathered relevant publications using reference snowballing. We included published records that were written in English and applicable to the design of health materials.

**Results:**

We found 40 articles that were appropriate for inclusion. Various instantiations of fluency have a uniform effect on human judgment: fluently processed stimuli generate positive judgments (e.g., liking, confidence). Processing fluency is used to predict the effort needed for a given task; accordingly, it has an impact on willingness to undertake the task. Physical perceptual, lexical, syntactic, phonological, retrieval, and imagery fluency were found to be particularly relevant to the design of health materials.

**Conclusions:**

Health-care professionals should consider the use of a perceptually fluent design, plain language, numeracy with an appropriate degree of precision, a limited number of key points, and concrete descriptions that make recipients imagine healthy behavior. Such fluently processed materials that are easy to read and understand have enhanced perspicuity and persuasiveness.

## Background

Health information is an important understanding resource for the general public: it enables them to engage in the management of their health condition and improve their well-being. In particular, written health information has a number of advantages, such as reusability, portability, and flexibility of delivery [1]; this information has a positive impact on the effectiveness of patient education [2]. However, a significant concern is that users may not obtain optimal benefits from health information because of their limited health literacy, i.e., the ability to comprehend the information and use it to make appropriate decisions about their health and health care [3]. Such resources as patient educational materials and public health information are often written at a readability level that is too high for most intended recipients [4]. In such cases, more information may cause target subjects to feel confused and powerless rather than empowered. Furthermore, health information needs to prioritize uninterested, resistant users as targets [5]. Accordingly, written health materials have to be readable and generate favorable evaluations of the message.

The study of processing fluency (PF) has become increasingly popular over the past decade. PF is defined as the inferred subjective ease with which new external information can be processed [6]. Studies have shown that fluently processed stimuli generate positive judgments (e.g., liking, confidence). For example, Song and Schwarz [7] demonstrated that with easily readable exercise instructions, participants were more willing to incorporate such exercises as part of their daily routines than when the instructions were difficult to read. Accordingly, researchers have considered PF in terms of readability as a generator of positive judgments. Sharing the implications of PF among health-care professionals would appear to be beneficial in producing effective health materials. Hitherto, however, no study has reviewed PF publications with respect to their potential application in writing health information.

The present review aimed to explore the implications for designing effective health materials from the perspective of PF. We conducted a systematic literature review to answer a research question: what instantiations of PF are applicable to the design of health materials? We suggested practical implications for designing health materials based on the results.

## Methods

To identify relevant studies, we searched seven online databases using “processing fluency” as a key word. The search encompassed both databases with a medical orientation and those with a focus on social sciences: MEDLINE, CINAHL, PsycArticles, PsycINFO, Communication Abstracts, Business Source Complete, and ERIC. The initial database search was conducted in early December 2015. The search yielded 538 articles published between 1980 and 2015. To assess their relevance, we applied a strict set of inclusion and exclusion criteria. For inclusion, records (journal articles, books and chapters) had to be published—either online or in print. We excluded all poster presentations and proceedings papers. Only records written in English were included. Regarding content, publications were deemed relevant and included when they explicitly discussed PF and when they generally had potential application to the design of health materials. We therefore excluded studies that used only priming manipulation, addressed only such topics as art and language learning.

**Fig. 1:**
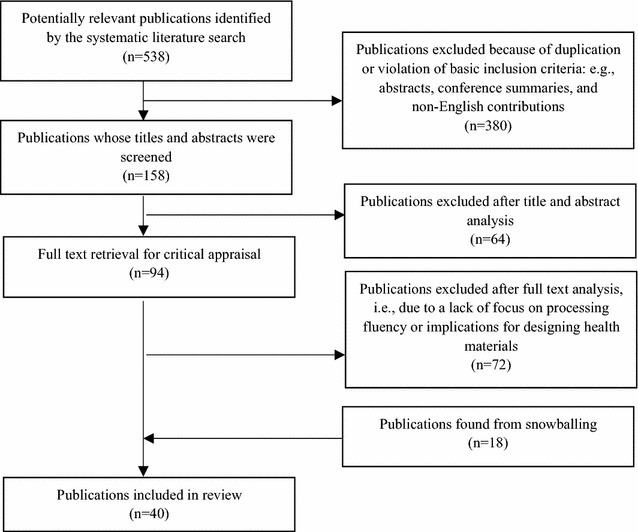
Analysis of the identified contributions

After the analysis of the titles and abstracts, we identified 94 unique publications as relevant. We subsequently analyzed the full text of each of the 94 articles. Following careful textual scrutiny, we excluded 72 publications due to a lack of focus on processing fluency or implications for designing health materials. We also gathered relevant publications using reference snowballing of the first 94 publications. After snowballing and full textual analysis, we added 18 articles. Thus, 40 articles were deemed fit for inclusion and subsequently review (Fig. 1).

## Results

### Study characteristics

Of the 40 included articles, 30 were experimental studies; 10 were review articles. Table 1 summarizes the 30 experimental studies. Most of the studies were in the fields of psychology, marketing, or consumer research. Only five studies were related to health care.

**Table 1:** Summary of experimental studies

Reference	Materials	Fluency	Main outcomes	Main results
Song and Schwarz [7]	Exercise instruction sheets, recipe materials	Clear or unclear font	Estimates of time and skill needed, task fluency, willingness to do the task	Participants reported that the behavior would take more time, would feel less fluent, and would require more skill. Therefore, they were less willing to engage in it, when the instructions were printed in a dysfluent font
Reber et al. [10]	Drawings	Matching or non matching prime, back ground contrast, presentation duration	Prettiness judgments	Perceptual fluency increased liking and the experience of fluency was affectively positive
Begg et al. [11]	Statements	Known or unknown names of source, familiar or unfamiliar statements	Truth judgments	Truth judgments were influenced by source recollection and statement familiarity
McGlone and Tofighbakhsh [12]	Aphorisms	Rhymed or unrhymed	Truth judgments	Rhymed aphorisms were judged to be more accurate
Alter and Oppenheimer [13]	Currency	Familiar or unfamiliar	Purchasing power judgments	Familiar forms of currency were perceived to have greater purchasing power
Brown et al. [14]	Faces	Familiar or unfamiliar	Credibility judgments	Repeatedly encountered familiar faces were judged to be more credible
Song and Schwarz [15]	Names of ostensible food additives and amusement-park rides	Easy or difficult to pronounce	Safety judgments	Products were judged to be riskier when their names were difficult to pronounce
Dreisbach and Fischer [21]	Number words	Clear or unclear font, high or low background contrast	Response times and error frequencies	Low processing fluency was not only used for effort prediction but also for effort adjustments
Gmuer et al. [22]	Labels of wine	Clear or unclear font	Judgments of taste	Wine in a bottle with a fluent font label was preferred over the same wine in a bottle with a dysfluent label
Guenther [23]	Syllabus	Clear or unclear font	Forecasted grade and course difficulty	Participants forecasted higher grades and estimated the course as easier after reading the fluent syllabus
Reber and Schwarz [24]	Statements	High or low background contrast	Truth judgments	Highly visible statements were more often judged to be true
Mosteller [25]	Product information	Clear or unclear font, high or low background contrast, low or high information density	Cognitive effort, positive affect, choice satisfaction	Fluent information resulted in less cognitive effort perception, greater enjoyment and greater choice satisfaction
Oppenheimer [26]	Essays	Complex or simple words and sentences, clear or unclear font, high or low visibility	Acceptance decisions of the applicant, perceived author intelligence	Complexity and dysfluency led to negative evaluations
Lowrey [27]	Product advertisement	Simple or complex syntactic	Recall, attitudes toward the brand, level of involvement	Syntactic complexity affected recall and persuasiveness of advertising
Miller [28]	Financial reports	High or low readability	Trading activity	More readable financial disclosures were associated with greater trading activity
Rennekamp [29]	Financial reports	High or low readability	Stock valuations, management competence and trustworthiness	A fluent report generated stronger reactions to both good and bad news
Tan et al. [30]	Financial reports	High or low readability	Judgments on the firm’s future performance	High readability improved understanding of the firm’s performance
Laham et al. [31]	Names of individuals	Easy or difficult to pronounce	Judgments of liking, positions in the firm hierarchy	Those who had phonologically fluent names were liked more and occupied higher status positions in firms
Dohle and Siegrist [32]	Names of medications	Easy or difficult to pronounce	Judgments of safety, effectiveness, side effects, and willingness to buy	Phonologically fluent medications were judged to be safer and to have fewer side effects; greater willingness to buy
Manley et al. [33]	Recruitment sheets for a medical study	Easy or difficult to pronounce, clear or unclear font	Judgments of attractiveness, complexity, expected risk and required effort	Participants judged the study more complex when they read a dysfluent sheet
King and Janiszewski [35]	Numbers, advertisements with numbers	Numbers from common arithmetic problems or not	Judgments of liking, product choices	Participants preferred numbers from common arithmetic problems more than other numbers
Coulter and Roggeveen [36]	Prices	Numbers constitute an approximation sequence or not, Numbers are multiples of one another or not	Purchase intentions and judgments of liking	When the numbers constituted an approximation sequence or were multiples of one another, incidences of price promotion predilection increased
Schwarz et al. [37]	Assertive or unassertive behaviors	Easy or difficult to recall	Self-rating of assertiveness	Higher retrieval fluency of assertive behaviors led to higher self-rating of assertiveness
Rothman and Schwarz [38]	Risk factors for heart disease	Easy or difficult to recall	Vulnerability judgments	Higher retrieval fluency of risk-increasing factors led to higher vulnerability judgments
Chang [39]	Product information	Easy or difficult to recall	Quality and monetary sacrifice judgments	Higher retrieval fluency led to higher evaluation of product quality and to less focus on monetary sacrifice
Wånke et al. [40]	Product information	Easy or difficult to recall	Brand evaluations	Higher retrieval fluency led to higher brand evaluation
Wänke and Bless [41]	Advertisements	Easy or difficult to recall	Product evaluations	Higher retrieval fluency led to higher product evaluation
Petrova and Cialdini [42]	Advertisements	To image or not	Brand attitudes and purchase intentions	Service was preferred more when the participants vividly imagined it
Mandel et al. [43]	Story	Plausible or implausible	Predicted future income	Participants who read a plausible success story judged they themselves can success more in future
Gregory et al. [44]	Service solicitation	To image or to be explained	Contract ratios	Subjects who imagined the benefits of a service were more likely to subscribe to the service than subjects who were merely given information

### Nature of PF and its effects

Every cognitive task, such as reading health materials, can be described along a continuum from effortless to highly effortful. This cognitive effort produces a corresponding metacognitive experience, which ranges from fluent to disfluent. Human judgment is influenced not only by the information content but also by the metacognitive experience of processing that information [8, 9]. For example, health materials that are written with familiar, simple words are easy to read and understand, and therefore assumed to be fluently processed, i.e., they possess lexical fluency. In contrast, materials that include a number of unfamiliar jargon terms are hard to read and understand, and therefore assumed to be disfluently processed. There are various types of fluency, as discussed below (e.g., syntactic, phonological, and retrieval fluency; for a review, see [6]); however, their effect on judgment is uniform: fluently processed stimuli generate a positive judgment among recipients [6]. More precisely, compared with less fluent stimuli, fluently processed stimuli are generally rated as follows: more pleasing [10]; more trustworthy [11, 12]; more valuable [13]; more honest and sincere [14]; and safer [15]. Fluently processed stimuli also have numerous other favorable attributes (for reviews, see [6, 16]). The manner in which the PF effect works has been explained by the hedonic marking hypothesis [10, 17], attributional accounts [18], and naive theories [19]. Recent studies have focused largely on naive theories to account for context-specific interpretations of the PF effect (for reviews, see [16, 19]).

Processing fluency also influences a recipient’s willingness to undertake a given task. The experience of fluent processing has been repeatedly demonstrated to serve as an affective signal. In general, high PF generates a positive affect, whereas low PF produces a negative affect [17]. The positive affect is a source of positive attitude, which is an antecedent to behavioral intention [20].

Processing fluency is used to predict the effort necessary for a given task; accordingly, it has an impact on willingness to do a task (discussed below) [21]. More precisely, low PF serves as an aversive signal and reduces willingness to engage in the given task [21]. For example, health materials that are presented in a small, hard-to-read font and use jargon terms and complex syntax cannot be processed fluently. Consequently, readers will suppose that greater effort is needed for the given task and are more likely to resist the health message—partly because of disfluency. The reverse also applies.

### Instantiations of PF

There are various instantiations of fluency (for a review, see [6]); however, the present review focuses on perceptual, lexical, syntactic, phonological, retrieval, and imagery fluency because these are particularly relevant in the design of health materials. We present instantiations of PF with experimental studies and then address their implications in designing health materials in the “Discussion”.

#### Physical perceptual fluency

The subjective ease with which stimuli are physically perceived (e.g., a clear or unclear font and the contrast between lettering and the background) is termed physical perceptual fluency [6]. Font manipulation is a common method that has been used to investigate the fluency effect. Gmuer et al. [22], for example, demonstrated that a label’s PF influenced taste evaluations: wine in a bottle with a label printed in a fluent font (Arial Narrow) was liked more than wine labeled with a disfluent font (Mistral) despite the fact that both the wine and label descriptions were identical. This study is an apt example of fluently processed materials’ effect of increasing judgments of liking.

A study by Song and Schwarz [7] has particularly strong implications for designing health materials. Participants were asked to read identical instructions for an exercise printed in an easy-to-read (12-point Arial) or difficult-to-read (12-point Brush) font. As predicted, they estimated that the exercise would take less time and felt quicker and more fluid when the font was easy to read than when it was difficult. Accordingly, participants also indicated greater willingness to make the exercise part of their daily routine when the font was easy to read.

A similar effect was obtained in a study using the syllabus of an undergraduate college course [23]. Participants read an identical summary syllabus printed in an easy-to-read (12-point Arial) or difficult-to-read (12-point Brush) font. As anticipated, participants believed they would receive higher grades and that the course would be easier when they read the more fluent font.

These two studies support experienced fluency of written materials as a subjective indicator of the effort needed for a particular task. If materials are easy to read and fluently processed, the perception of the required effort decreases and recipients may have a greater willingness to undertake the task [21].

Variations in the contrast between the text and background are also frequently used in fluency research. In a classic study by Reber and Schwarz [24], statements of the form “Town A is in country B” (e.g., Osorno is in Chile) were presented at the center of a computer screen. Half of the statements were true, and half were untrue. The visibility of the statements was manipulated by contrasting the colors against the white background. Highly visible colors included blue and red; moderately visible colors included green, yellow, and light blue. The participants judged the highly visible statements as being more probably true than statements with low visibility.

A recent study manipulated the font, background contrast, and information density on a fictitious online shopping Web site [25]. The product information appeared in Arial or Mistral font, with a white or gray background against black text; five or 15 attributes were presented for each alternative on fluent and disfluent pages. Fluently processed pages resulted in less cognitive effort perception, greater enjoyment of the task, and consequently greater choice satisfaction among participants.

In sum, high perceptual fluency (e.g., using an easily legible font with high background contrast) increases the likelihood of positive judgments and enhances recipients’ willingness to do a given task.

#### Lexical, syntactic, and phonological fluency

The subjective ease with which words are processed is termed lexical fluency; the subjective ease of parsing grammatical constructions is termed syntactic fluency; and the subjective ease of pronunciation is termed phonological fluency [6]. Collectively, these are referred to as linguistic fluency [6].

In general, highly readable materials are lexically and syntactically fluently processed; consequently, they generate more favorable evaluations of the message [26, 27]. Miller [28] measured the readability of financial reports of firms using readability measures. The results showed that more readable financial disclosures were associated with greater trading activity among small investors. Along similar lines, Rennekamp [29] adapted financial disclosure materials from those of a real company. Rennekamp created more readable and less readable versions of a financial report by manipulating such factors as sentence length, simplicity of terms, and ease of syntax. After reading the fluent or disfluent financial reports, participants were asked to estimate the appropriate common stock valuation, management competence, and trustworthiness of the firm. The fluent report generated stronger reactions: judgments were more positive to good news and more negative to bad news. Additionally, participants were more likely to feel they could trust a fluent report. A similar result is reported by Tan et al. [30]. Thus, highly readable materials generate more favorable evaluations and stronger reactions.

The phonological ease of words also influences our judgments. Certain letter strings are easier to process than others. For example, English speakers cannot naturally pronounce the string “SBG” (disfluent), whereas they can easily pronounce the equally nonsensical “SUG” (fluent). Laham et al. [31] showed that people form more positive impressions of easily pronounceable names and their bearers (e.g., Mr. Smith vs. Mr. Colquhoun). They further revealed that people with easily pronounceable names occupied higher-status positions in firms. Dohle and Siegrist [32] found that easily pronounceable medications were perceived to be safer and have fewer side effects, and participants expressed greater willingness to buy them than medications that were difficult to pronounce. Thus, phonologically fluent words generate favorable evaluations [33].

Round numbers are also processed with linguistic fluency. Such round numbers as 10, 25, and 100 are frequently used because they can express approximate quantities and therefore refer to an entire range of magnitudes [34]. For example, one might refer to any given number within the range 90–110 (e.g., 93, 99, 102, and 106) as “about 100.” Accordingly, the number 100 is used more frequently and processed more fluently than those other numbers, leading to greater liking [34, 35]. Coulter and Roggeveen [36] investigated whether numbers that were members of a base-10 approximation sequence were preferred in a shopping context. The authors showed that prices and discount amounts that were members of an approximation sequence (e.g., $100, $30, $70, and 70% discount) generated greater liking and purchase intentions than did precise prices and discount amounts (e.g., $101, $29, $72, and 71%). Thus, round numbers generate more positive judgments.

#### Retrieval fluency

The subjective ease with which individuals recall information or retrieve arguments relevant to a message is termed retrieval fluency [6]. Although the assumption of retrieval fluency has enjoyed great popularity since Tversky and Kahneman [8] introduced availability heuristic, it was not adequately tested before the 1990s [37]. Rothman and Schwarz [38] asked participants to recall either three or eight behaviors that could increase or decrease their risk for heart disease. Although recalling three risk factors was relatively easy, recalling eight risk factors was difficult. Participants without a family history of heart disease reported lower vulnerability after recalling eight rather than three risk-increasing behaviors and higher vulnerability after recalling eight rather than three risk-decreasing behaviors. They presumably used a heuristic judgment strategy that relied on the ease of recall: if it is easy (difficult) to recall, it is likely (unlikely) to occur. In a similar vein, information that is easy to retrieve has been demonstrated to increase the favorable evaluation of a product [39, 40] as well as the persuasiveness of an argument [41].

#### Imagery fluency

The subjective ease with which one can imagine hypothetical scenarios that have not yet occurred is termed imagery fluency [6]. Brand attitudes and purchase intentions become increasingly positive when individuals can vividly imagine the product [42]. Business school students were able to imagine success more easily and judge that they themselves were more likely to succeed in the future when they read a story about a plausible (rather than implausible) level of success [43]. Gregory et al. [44] showed that subjects who imagined themselves enjoying the benefits of a cable TV service were more likely to subscribe to the service than subjects who were merely given information about the service. Thus, self-generated images of future behavior and events have an effect on subsequent judgments and behavior.

## Discussion

Studies have shown that evaluating information and making decisions depend not only on the presented information itself but also on the information’s PF. As described in this review, high PF of information generates positive judgment among recipients. The implications of this cast new light on the importance of designing readable health materials. Traditionally, health materials have been difficult to process because of excessive jargon [45, 46], excessively high reading levels [4], and dense presentation of information [47, 48]. In recent years, low health literacy has been recognized as one of the barriers to understanding and using health information in making appropriate decisions about one’s health and health care [3]. It is also recognized that health literacy is a result of interactions between an individual’s skill and the demands of the society in which the individual lives, including the manner in which health information is communicated [49]. Accordingly, in recent years, efforts to make health information easy to read have become recognized as more important. However, relevant efforts in health care have focused on lowering the barriers to health information for those with low literacy skills. In contrast, as indicated in this review, fluency research has the active goal of focusing on increasing positive judgments and enhancing behavioral willingness. Studies of PF indicate that easiness to read, recall and imagine (i.e., high PF) can contribute to enhancing both the perspicuity and persuasiveness of health materials.

Based on this review, we suggest five practical implications for designing effective health materials that generate positive judgment and enhance recipients’ willingness to follow healthy behavior.

### Design materials with readability: physical perceptual fluency

Assessment tools for written materials propose guidelines regarding fonts and type size to make materials easy to read [50, 51]. Doak et al. [50] states that type size and fonts can make text easy or difficult for readers at all skill levels, and they recommend that text be in a serif font in 12 point or larger. That study also recommends a good contrast between the text and background [50]. As indicated in the “Results” section, high physical perceptual fluency (e.g., an easily legible font with high background contrast) increases positive judgments and enhances recipients’ willingness to do a given task [7, 21–25]. Health materials should be designed with perceptual fluency to generate positive judgments among recipients.

### Use of plain language: linguistic fluency

Without exception, assessment tools and guidelines for health materials emphasis the importance of plain language; i.e., using common and familiar words, short sentences, and explicit sentence constructions, so that readers can process the information more easily and quickly [50, 52–55]. As shown in the “Results” section, phonologically fluent (i.e., easy to pronounce) words generate favorable evaluations [31–33]. Furthermore, highly readable materials that are written with plain language generate more favorable evaluations, greater trust and stronger reactions [26–30]. Although these PF studies were in contexts other than health, health materials also should generate favorable evaluations and trust, as well as strong reactions from their recipients, especially in emotional appeals [56]. Favorableness, trust and strong reaction are sources of persuasiveness [57]. Using plain language that is linguistically fluently processed is considered to be important not only for making written health materials comprehensible but also for enhancing persuasiveness of those materials.

### Use round numbers: linguistic fluency

Numerical precision is needed for analysis and reporting in a scientific research context. However, unnecessary numerical precision is undesirable and can be misleading to laypeople, such as patients. Ehrenberg [58] points out that recipients can deal effectively only with numbers that contain no more than two significant digits. Assessment instruments for written materials therefore recommend the deletion of unnecessary numbers and suggest that when numbers are used, they should be clear and easy to understand [53, 54]. As indicated in the “Results” section, round numbers generate more positive judgment than precise numbers [34–36]. Lang and Secic [59] recommend that numbers in health materials should be rounded unless greater precision is actually necessary—and then reported with the appropriate degree of precision.

### Limit number of key points: retrieval fluency

It is widely accepted that there is a general limit on human cognitive capacity [60]. Humans are “cognitive misers” [61] and conserve cognitive resources. Thus, people attempt to minimize the cognitive workload according to the law of least mental effort [62]. Accordingly, when patients or caregivers are overwhelmed with too much health information, their ability to comprehend, recall, and use this information can decline [63]. Nevertheless, written health materials tend to be information dense [47, 48]. Kaphingst et al. [48] reviewed health materials and found that only half limited the number of key points to five or fewer per section. When health materials are information dense, recipients generally have difficulty in memorizing them and recalling the content. As demonstrated in the “Results” section, the ease of recall of information influences judgment [37–41]. Furthermore, memory is an antecedent of behavior: information has to be memorized and recalled if a recipient is to perform the health behavior indicated [64]. Accordingly, as Brega et al. [55] suggest, health-care professionals should prioritize what needs to be discussed and limit information to three to five key points so that the content may be easily processed and recalled.

### Make recipients imagine health behavior: imagery fluency

As shown in the “Results” section, self-generated images of future behavior and events influences subsequent judgments and behavior [42–44]. One of the purported reasons for the imagery fluency effect is that it makes images of relevant behavior subsequently more available in memory and consequently makes them appear more probable [8, 65]. Image generation and memorability are prompted by concrete descriptions [66]. As Shoemaker et al. [53] recommend, health behavior described in health materials should be broken down into manageable, explicit steps and portrayed concretely. In this way, recipients can easily imagine the behavior, which will help them do likewise.

### Limitations

Although the findings described in this review provide a promising starting point for further research into the PF effect in a health-care context, some limitations should be considered. It may be assumed that we retrieved the most important contributions dealing with PF using our strategy of combining a database search with an extensive manual search; however, we may still have missed some publications. In addition, most of the contributions detected in the search were studies in areas other than health. The applicability of findings from outside health care to a health-care context deserves careful consideration. Future studies need to investigate the fluency effect in the health-care context. Furthermore, some recent studies have focused on moderating variables of the PF effect, such as construal level and need for cognition [67–69]. Additionally, recent studies have investigated the cases in which processing disfluency was efficacious [70–73]. These should be noted and explored to better our understanding of the PF effect.

## Conclusions

When health-care professionals design health materials, they should consider perceptually fluent design, plain language, numeracy with the appropriate degree of precision, a limited number of key points, and concrete descriptions so that recipients may imaging the health behaviors. Doing so will lower the literacy level of health materials and generate positive judgments, and it may enhance recipients’ willingness to perform healthy behavior. If we design health materials to be processed more fluently, we will be able to motivate recipients more effectively.

## Abbreviation

PF: processing fluency
